# Designing an evaluation tool for evaluating training programs of medical students in clinical skill training center from consumers’ perspective

**DOI:** 10.1186/s12909-024-05454-7

**Published:** 2024-05-09

**Authors:** Rezvan Azad, Mahsa Shakour, Narjes Moharami

**Affiliations:** 1 Medical Education development center, Arak University of Medical Sciences, Arak, Iran; 2 Medicine School, Arak University of Medical Sciences, Arak, Iran

**Keywords:** Program evaluation, Scriven, Medicine, Consumer-oriented, Clinical skills lab

## Abstract

**Introduction:**

The Clinical Skill Training Center (CSTC) is the first environment where third year medical students learn clinical skills after passing basic science. Consumer- based evaluation is one of the ways to improve this center with the consumer. This study was conducted with the aim of preparing a consumer-oriented evaluation tool for CSTC among medical students.

**Method:**

The study was mixed method. The first phase was qualitative and for providing an evaluation tool. The second phase was for evaluating the tool. At the first phase, after literature review in the Divergent phase, a complete list of problems in the field of CSTC in medicine schools was prepared. In the convergent step, the prepared list was compared with the standards of clinical education and values of scriven. In the second phase it was evaluated by the scientific and authority committee. Validity has been measured by determining CVR and CVI: Index. The face and content validity of the tool was obtained through the approval of a group of specialists.

**Results:**

The findings of the research were in the form of 4 questionnaires: clinical instructors, pre-clinical medical students, and interns. All items were designed as a 5-point Likert. The main areas of evaluation included the objectives and content of training courses, implementation of operations, facilities and equipment, and the environment and indoor space. In order to examine the long-term effects, a special evaluation form was designed for intern.

**Conclusion:**

The tool for consumer evaluation was designed with good reliability and trustworthiness and suitable for use in the CSTC, and its use can improve the effectiveness of clinical education activities.

## Introduction

Mastering clinical skills is one of the essential requirements for becoming a physician and pre-clinical courses play an important role in forming these clinical skills in medical students. The importance of these courses is such that a Clinical Skill Training Center (CSTC) has been formed especially for this purpose, which is nowadays used for training pre-clinical skills and some of the more advanced procedures such as operating room simulation [1]. The CSTC is an educational environment where students can use the available resources and the supervision of experienced faculty members to be introduced to clinical skills, train and gain experience in these skills and receive immediate feedback to resolve their mistakes and shortcomings [2]. The aim of the student’s participation in this center is the training of students who have sufficient theoretical knowledge but lack the necessary skills for working in the clinical setting. Therefore, this center supports students in the acquisition, maintenance and improvement of their clinical medical skills [3]. In this center, students can learn and repeat treatment procedures in a safe environment without severe consequences which reduces their stress and allows them to train and learn [4]. In this study, medical students attend this center for the first time after the end of theoretical course and before entering the hospital for the first time and Preliminary learn practical medical skills such as performing a variety of examinations and history taking. Then, in externship and internship, they can practice more advanced courses such as cardiopulmonary resuscitation, dressing and stitches etc. in small groups.

The importance of these centers like CSTCs is the fact that learning a large number of practical and communicational skills related to theoretical knowledge is one of the essential characteristics of medical education and can play an important role in the future careers of the students and training of specialized human resources in the field of medicine and healthcare [4]. However, one of the important matters in clinical training is the quality of education which can directly affect the quality of healthcare services provided to society. The quality of education is, in turn, affected by the details of the educational programs. Therefore, the evaluation of educational programs can play an important role in providing quality equations. In other words, using suitable evaluation mechanisms creates the requirements for performance transparency and accountability in the clinical education system in medical education [5]. Observing the principles of evaluation can also help determine the shortcomings and programs in educational programs [2]. However, the evaluation of educational programs is often faced with difficulties. Evaluations conducted to ensure the suitable quality of education for medical students must determine whether the students have achieved acceptable clinical standards which is only possible through careful evaluation of their training programs [2].

There are various problems concerning evaluation tools. The faculty members in medicine are still faced with challenges concerning the improvement of evaluation tools and the creation of tools for evaluating factors which are hard to quantify or qualify, such as professionalism, group work and expertise [6]. 

Despite various theories regarding evaluation, the lack of credible and valid evaluation tools for educational programs is still being felt [7]. Using suitable evaluation tools can create an overview of the current situation of the training programs based on the quality factors of the curriculum and can be used as a guideline for decision-making, planning, faculty development and improving the quality of education [8]. Perhaps the most important value of a suitable evaluation tool for training programs is providing a clear picture and operational and measurable measures regarding the implementation of educational programs. Furthermore, after completion, such a tool can be used as a constant interventional screening tool by academic groups, faculty members and authorities in practical training programs.

The consumer-oriented model advocated by evaluation expert and philosopher Michael Scriven. This model of evaluation like other models, is to make a value judgment about the quality of a program, product, or policy in order to determine its value, merit, or importance, but in this model, the value judgment is based on the level of satisfaction and usefulness of the curriculum for the consumers of the program. It is achieved and the evaluator considers himself to be responsive to their needs and demands. The models that are included in this approach have paid more attention to their responsibility towards the consumers of curriculum and educational programs.it is an exercise in value-free measurement of whether program goals were achieved [9, 10].

The current study aims to design an evaluation tool for training programs in the CSTC based on consumers’ perspectives and assess its validity and reliability to facilitate the evaluation of educational programs and help improve the practical skills of medical students. Therefore, the prepared evaluation tool not only can be used for continuous improvement of educational equality but can also be used for validation of educational programs.

## Subjects and methods

The study was mixed method with triangulation approach. This was a developmental study for developing an evaluation tool for educational programs of the CSTC in medicine schools from consumers’ perspective using data gathered through qualitative study, descriptive – survey study and from many resources. The study was done in 2020 until 2022 and in Arak University of Medical Sciences. Samples were students in different level, and clinical teachers who are consumers and main stakeholders. This study included two main phases.

The first phase was qualitative. Samples were literature and 10 experts. Sampling was purposeful. This phase was for decision-making regarding factors used for evaluating the educational programs of the CSTC. In this phase and to create a deep understanding of the topic, the literature related to the subject matter was reviewed. The reviewed literature related to evaluation was based on the consumers’ perspective evaluation and questionnaire preparation method. Then, using the Scriven consumer opinion questionnaire, standards for CSTC, and the available literature, interviews were conducted with experts and stakeholders in the CSTC. These interviews aimed to prepare a comprehensive list of problems, and concerns related to the educational programs at the clinical skill training center which the evaluation tool aimed to answer. This stage was known as the divergent stage where the topics discussed in the interviews included educational goals, content, equipment, educational processes, the environment and physical location. Some of the questions asked in this stage included “What is the level of achieving educational goals among students in the current program?”, “How effective is the practical program of the center in improving the clinical skills of the students?”, “Does the center has access to sufficient tools and equipment for completing its educational program?” and “what are the long-term effects of CSTC’s educational program?”

In the next step, known as the convergent step, the list prepared in the previous stage was combined with the educational standards for CSTCs provided by the deputy of education, ministry of health as well as Scriven criteria. The results were then carefully assessed by a scientific and authority committee consisting of the Educational Deputy of Clinical Education of the Faculty of Medicine, Director of Educational Affairs of the Faculty of Medicine, Director of Clinical Skills Training Center and Curriculum, Expert of Clinical Skills Center and Bachelor of Technical Affairs of Clinical Skills Training Center in the Faculty of Medicine of Arak University of Medical Sciences. The questionnaire items were selected based on the importance and evaluation criteria. The data gathering tool was prepared after determining the evaluation questions, data gathering sources and designing the evaluation method. Customers in this study were clinical training faculty members and medical students (externship, pre-clinical and internship students). Therefore, we designed four questionnaires with special questions. Every questionnaire is designed in 5 domains (Learning objectives and course content, Equipment and tools, Educational processes, Environment and physical location).

The second phase was quantitative and it was survey. Samples were professors and who were experts in subject and medical students (externship, pre-clinical and internship students). Sampling was conventional and purposeful. 10 faculty members and 71 students were selected. This phase was for measuring the questionnaire’s face and content validity. The validity was measured using Content Validity Ratio (CVR) and Content Validity Index (CVI) using Lawshe’s method. In this method, the opinion of experts in the field concerning the questionnaire content is used to calculate these factors [11]. A total of 10 faculty members participated in the validity survey and including faculty members from specialty fields of medical education, gynecology, infectious diseases, emergency medicine, pediatric medicine, nursing and midwifery. After explaining the research goals to the participants and providing them with the operational definitions related to the contents of the items, they were asked to mark each item in a table using a three-part Likert scale using “essential”, “useful not nonessential” and “nonessential” scores. Then, Content Validity Ratio was calculated using the following equations. **CVR=**$\frac{\varvec{n}\varvec{e}-\varvec{n}/2}{\varvec{n}/2}$**.** In this equation, n is the total number of experts, and n_e_ is the number of experts who have selected the “essential” score. Using the CVR table, the minimum CVT value for accepting an item based on the participants’ opinions was set at 0.62.

After calculating CVR, the method proposed by Waltz & Bausell was used for determining the CVI. To this end, a CVI evaluation table was prepared for the items using a four-part scale including “unrelated”, “requiring major revision”, “requiring minor revision” and “relevant” scores and delivered to the 10 participating experts who were asked to provide their opinions regarding each item. Then, the CVI value was calculated for each item by dividing the total number of “requiring minor revision” and “relevant” answers by the total number of experts. The items with CVI values higher than 0.79 were accepted [11, 12]. The reliability of the questionnaire was determined with emphasis on internal correlation with the help of SPSS software and was higher than 0.8, which confirmed the suitable reliability of the questionnaire. A panel of experts then conducted a qualitative review of the items, edited their grammar, and modified unclear statements based on the research goals. In general, the entire phrase should have been accepted by the majority of the panel based on simplicity, clarity and lack of ambiguity. The face validity was also calculated by scoring the effect of each item on the questionnaire. This score was then used to eliminate phrases with scores lower than 1.5. After evaluating the face validity, Content Validity Ratio (CVR) was calculated by the experts and items with CVR values less than the threshold value were selected and eliminated. After that, we used this tool by 71 students and 11 teachers to assess reliability according to Cronbach’s alpha.

## Results

The results of the current study indicate that according to the faculty members and experts participating in this study, the evaluation of educational programs of clinical skill training centers includes evaluation of programs in regards to goal and content, educational processes, equipment and tools, and environment and physical location. After interviews with clinical training experts and a review of relevant literature, 4 separate questionnaires were developed for clinical training faculty members, pre-clinical students, internship students, and externship students. All experts as samples answered all questions for validity and 71 students of 90 students completely answered the questionnaires.

The questionnaire for faculty members included 35 items (Table 1), the one for interns included 6 items (Table 2), the externship students’ questionnaire included 29 (Table 3) items and the questionnaire for pre-clinical students included 41 items (Table 4). All items were designed for scoring using a 5-point Likert system (very low, low, average, high, very high).

**Table 1:** Items of questionnaire for clinical faculty members

Domains	Items
Goal and content	• History taking • Physical examination • Procedural skill • Basic resuscitation • Advanced resuscitation • Executive skills • Prescribing and patient management skills
Equipment and tools	• The quality of the examination tool • The number of special examination tools compared to the number of students in each workshop • The quality of specialized mannequins • Suitability of the mannequin with the subject of education • Quality of audio-visual facilities • The quality and quantity of ICT facilities • The quality and quantity of closed-circuit imaging camera for control and observation of training
educational processes	• The length of time allocated to each session in CSTC • Regular attendance of pre-clinical medical students (minimum one time per month) • Attendance frequency of medical externs (minimum one time in two weeks) • The quality of information and the coordination of the center with the professors • The quality of service provided by the center’s expert to provide office services • The quality of service of the CSTC for the use of mannequins and audio-visual equipment • The quality of using Simulated Patient(SP) • The positive effect of training students in the CSTC on teaching in the real clinical environment • The effect of training in clinical skills center on increasing interest and motivation to teach clinical subjects
Environment and physical location	• Easy accessibility • Flexibility in use • Similarity to the actual bedside environment • The quality of the space for displaying skills • The quality of students’ practice space • Space for using educational multimedia • Teachers’ rest and preparation space • Dedicated work space (a room equipped with a desk and computer)

**Table 2:** Items of questionnaire for internship students

Domains	Items
Long-term effects of training courses	• Matching practical training programs with educational needs • The effect on the development of basic skills (such as procedural, diagnostic and clinical examination skills) • The effect of the center’s equipment and educational facilities on bedside performance • The effect of the space and physical environment of the center on their performance in hospital • Effect on increasing interest in bedside work • The effect of reducing the stress of being at the bedside

**Table 3:** Items of questionnaire for externship students

Domains	Items
Learning objectives and course content	• Procedural skill • Basic resuscitation • Advanced resuscitation • Executive skills such as issuing and death certificate • Prescribing and patient management skills
Equipment and tools	• Time and quality of having a procedural mannequin • Time and quality of having a basic resuscitation mannequin • Time and quality of having an advanced resuscitation mannequin • The quality of information technology facilities • Quality of audio-visual facilities
Educational processes	• The time of each skill training session at the clinical skills center • The need to attend • Clear explanation of practical skills in each workshop by professors • Teaching in workshops with a variety of methods except lectures • The teacher supervises the performance and gives feedback • Use of new evaluation methods • The quality of using Simulated Patient(SP) • The effect on increasing your interest and motivation to learn clinical subjects • The quality of service provided by the center’s expert to provide office services • The service quality of the center’s technician for teaching the use of mannequins and audio-visual equipment
Environment and physical location	• easy accessibility • Similarity to the actual bedside environment • Access to a dedicated space to practice skills and receive feedback • Space for using educational multimedia

**Table 4:** Items of questionnaire for pre-clinical students

Domains	Items
Learning objectives and course content	• History taking • Vital sign checking • urology examinations • neurology examination • gynecology examination • Breast and abdominal examination
Equipment and tools	• Time and quality of having a breast examination mannequin • Time and quality of having a vaginal examination mannequin • Time and quality of rectal and prostate examination mannequin • Time and quality of having an abdominal examination mannequin • Time and quality of having a fundoscopy mannequin • Time and quality of having a stethoscope • Time and quality of having a barometer • Time and quality of owning a reflex hammer • Time and quality of having an ophthalmoscope • The quality of information technology facilities • Quality of audio-visual facilities
Educational processes	• The time of each skill training session at the CSTS • Regular attendance of trainees • Clear explanation of practical skills expected in each workshop by professors • Teaching in workshops with a variety of methods except lectures • The teacher supervises the performance and gives feedback • Use of new evaluation methods • The quality of using Simulated Patient(SP) • The effect on increasing your interest and motivation to learn clinical subjects • The quality of office services • The quality of service of the training center for the use of mannequins and audio-visual equipment.
Environment and physical location	• easy accessibility • Similarity to the actual bedside environment • Access to a dedicated space to practice skills and receive feedback • Space for using educational multimedia

The face validity of questionnaires was evaluated using qualitative and quantitative approaches. Among 117 items in 4 questionnaires, 6 items didn’t have suitable content validity (CVR < 0.62) which were eliminated according to the following table (Table 5). 111 items had CVR ≥ 0.62 and the results of the CVI assessment indicated that all items were acceptable.

**Table 5:** CVR

No.	Clinical Faculty members’ Questionnaire	Pre-Clinical Students’ Questionnaire	Internship Students’ Questionnaire	Residency Students’ Questionnaire
Total Items	37	42	30	8
CVR ≥ 0.62	35	41	29	6
CVR < 0.62	2	1	1	2

The reliability of the questionnaires was investigated using Cronbach’s Alpha with emphasis on internal correlation with the help of SPSS software as presented in the following table, which confirms the reliability of the questionnaires (Table 6). The reliability in all questionnaire was more than %83. Therefore, all items received acceptable reliability and validity scores.

**Table 6:** Cronbach’s alpha

Questionnaire	Total Items	Cronbach’s Alpha
Residency Students	6	83%
Internship Students	29	97%
Pre-Clinical Students	41	93%
Clinical Faculty Members	35	97%

## Discussion

In the current study, a comprehensive researcher-made questionnaire was prepared based on the opinions of experts and curriculum designers while considering all relevant resources and literature which is a unique tool in Iran regarding the expansiveness of the scope. The prepared tool was then used to evaluate the activities of the clinical skills training center in 5 domains (1) program goals and content, (2) tools and equipment, (3) educational processes, (4) environment and physical location and (5) long-term effects of the curriculum.

The first part of the evaluation tool prepared in the current study aims to assess the *objective goals* of program according to the consumer’s views. CSTC is suitable for training basic and practical skills which are often neglected due to time constraints during the students’ presence in clinical environments [6]. The factors investigated in this area using the current tool included basic skills such as patient interview, basic resuscitation, clinical examination, practical clinical activities, interpretation of essential clinical findings, prescription skills and patient management. Other studies have also investigated similar factors. For example, Imran et al. (2018) in their study evaluated the attitude of students towards this center and stated that participation in Skill Lab sessions in the pre-clinical years will assist students in their clinical year to achieve better overall performance, as well as better communication skills and self-esteem [1]. According to previous studies, the majority of students preferred participation in pre-clinical straining in these centers due to the advantages of skill labs for learning clinical skills [3]. Another study showed that the majority of students prefer participation in skill lab for learning essential clinical skills such as venous blood sampling, catheterization, endotracheal intubation, listening to respiratory sounds, genital examination, etc. compared to directly performing these procedures on patients [2]. The designed tools in current study evaluated some of these learning objectives. But because of evaluating 5 domains and many questions in every domain, we summarized them to be user friendly. Every questionnaire had some question for objectives that questionnaire respondents as customers (faculty members and medical students) could reply them.

The second part of this evaluation tool is for assessing educational tools such as educational mannequins and models, medical examination devices (Stethoscope, sphygmomanometer, otoscope and ophthalmoscope), medical consumables, audio-visual equipment and information technology facilities. According to the studies, a common factor in CSTCs is access to a wide range of tools in each university as well as using updated technologies for education. These innovations have even resulted in the improved academic ranking of some colleges and medical universities in the world [12]. The quality of these educational tools is the other important item in many studies [13]. The quality for mannequin is depended to fidelity. Brydges et al. in his study showed that higher fidelity causes more learning and less time for learning. They suggested that clinical curricula incorporate exposure to multiple simulations to maximize educational [14].

The third part of this tool is educational processes consisted of evaluating factors such as the length and number of workshops, the effect of CSTC on teaching in a clinical environment, the effect of the center on increasing the motivation and interest in clinical topics, use of volunteer patients and actors and use of modern teaching and assessment methods. This area evaluates the educational process as an important part of clinical training. The importance of this area is also confirmed in other studies. CSTC enables students, including interns and new students, to practice procedures without fearing the consequences. Furthermore, there is also no time of ethical constraints in these practices, enabling the students to be trained in treatment procedures and physical examinations which can be dangerous or painful for the patient [2]. In this regard, the standardized patient is one of the popular methods used in universities around the world. For example, the University of Massachusetts had been using standardized patients as an education and assessment tool and even as clinical trainers for more than 20 years [8]. Another example is the simulation center of Grand Valley State University, which provides significant tools for the management of standardized patients, including registration and deployment of standardized patients as needed. This center has designed a website for the registration of standardized patients, which allows individuals to register based on certain criteria, before being trained and deployed according to the protocols [8].

The effectiveness of clinical skill training centers on motivation was presented in a study by Hashim et al. (2016) on the effects of clinical skill training centers on medical education. According to the results of this study, 84 to 89 per cent of students believed that these centers increase the motivation for medical education as well as interest in learning clinical skills [3]. In regards to the use of modern methods, one of the most recent examples is the use of clinical simulations using multimedia tools and software which can be used for improving psychological and psychomotor skills. Studies have shown that these centers also lead to improved motivation and independent learning tendencies among students [13].

The forth part, is related to the evaluation of the environment and physical location in the current tool, accessibility, flexibility in application, similarity to a real environment, specialized training spaces, receiving feedback and use of multimedia technologies. These factors are extracted according to the opinions of experts and stakeholders and have been used in similar studies. According to the standard for clinical skill training centers presented by the Ministry of Health, Treatment and Medical Education, the preferred physical location for a clinical skill training center includes a large area with a flexible application as well as a wardroom, nursing station, ICU or smaller rooms with specialized applications such as operation room and resuscitation room. Furthermore, a clinical skill training center must have access to a suitable location for providing students with multimedia education [8].

James et al. in their study, have shown effectiveness of an experimental pharmacology skill lab to facilitate training of specific modules for development of core competencies of parenteral drug administration and intravenous drip settings using mannequins for development of skills in administering injections for undergraduate medical students [15]. These factors were included in the evaluation questionnaire prepared in the current study. In the study by Hashim et.al.(2016), 62 participants believed that the time constraints and pressure of the clinical environment were not present in CSTC during learning clinical skills. Therefore, these centers can help students improve their skills by making them feel secure and resolve their concerns about the consequences of their actions. According to the students participating in this study, approximately 70 to 75 per cent of students felt more secure regarding mistakes and less worried about harming patients during clinical procedures after training clinical skills on mannequins available at clinical skill training centers [3].

The fifth part includes evaluating the long-term effects of education and evaluating the conformity between the center’s curriculum and educational needs, the effect of the center on improving essential skills, the effect of curriculum on interest, stress and facilitating clinical procedures. Ji He Yu et al. observed that after training in a clinical skill training center and simulations, students show a significantly lower level of anxiety and a significantly higher level of self-esteem compared to before the training. Furthermore, after experiencing the simulation, students without previous simulation experiences showed lower anxiety and higher self-esteem [16]. In a systematic review by Alanazi et al., evidence showed that participation in CSTC and using simulation can significantly improve the knowledge, skill and self-esteem of medical students [17]. Furthermore, a study by Younes et al. showed that adding a simulation program to a normal psychology curriculum improves the quality of education and the self-esteem of medical students [18]. In another study, Hashim et.al.(2016) showed a positive attitude among the students regarding the effectiveness of clinical skill training centers for improving skills, self-esteem as well as learning new clinical skills [3]. Therefore, based on the role of clinical skill training centers in improving the motivation and self-esteem of students presented in previous studies, these factors can be important in the evaluation of clinical skill training centers and therefore included in the evaluation questionnaire.

Limitations:

We had some limitations in our study. 1)There wasn’t any evaluation tool for evaluating Training Programs of medical students in Clinical Skill Training Center according to Consumers’ Perspective. Therefore, comparison was difficult and we compared every domain with results of other studies. The study was triangulation and we used many resources to designing this tool and it reduced biases. 2) In convergent step we extracted many items, but because of the possibility of non-response all questions, we couldn’t use all of them and questionnaires are summarized. To assuring no important item is neglected, experts in medical education checked the items.

## Conclusion

There are many items in an evaluation tool for evaluating the Clinical Skill Training Center from Consumers’ Perspective. Some of these items could be answered by some consumers not all of them. In this tool is defined in 4 tools for four type of consumers. In every tool respondent answer questions in 5 domains (Learning objectives and course content, Equipment and tools, Educational processes, Environment and physical location). The evaluation tool designed in the current study offers suitable reliability and validity and can be used for evaluating CSTC from consumers’ perspectives. The application of this tool can help improve the effectiveness of educational activities and the curriculum in clinical skill training centers.

## Data Availability

The datasets used and/or analyzed during the current study are available from the corresponding author upon reasonable request.

## Abbreviations

CSTC: Clinical Skill Training Center
CVR: Content Validity Ratio
CVI: Content Validity Index
